# Vine Water Deficit Impacts Aging Bouquet in Fine Red Bordeaux Wine

**DOI:** 10.3389/fchem.2017.00056

**Published:** 2017-08-02

**Authors:** Magali Picard, Cornelis van Leeuwen, François Guyon, Laetitia Gaillard, Gilles de Revel, Stéphanie Marchand

**Affiliations:** ^1^Unite de Recherche Œnologie EA4577, Institut des Sciences de la Vigne et du Vin, Université Bordeaux Villenave d'Ornon, France; ^2^Unite de Recherche Œnologie USC 1366, Institut des Sciences de la Vigne et du Vin, Institut National de la Recherche Agronomique Villenave d'Ornon, France; ^3^Unité Mixte de Recherche Ecophysiologie et Génomique Fonctionnelle de la Vigne, Ecole Nationale Supérieure des Sciences Agronomiques de Bordeaux-Aquitaine, Institut National de la Recherche Agronomique, Université Bordeaux Villenave d'Ornon, France; ^4^Service Commun des Laboratoires Pessac, France

**Keywords:** wine aging bouquet typicality, mint and truffle aromas, vine water status, water deficit, wine carbon 13 isotope, δ^13^C, vintage effect

## Abstract

The aim of this study was to investigate the influence of vine water status on bouquet typicality, revealed after aging, and the perception of three aromatic notes (mint, truffle, and undergrowth) in bottled fine red Bordeaux wines. To address the issue of the role of vine water deficit in the overall quality of fine aged wines, a large set of wines from four Bordeaux appellations were subjected to sensory analysis. As vine water status can be characterized by carbon isotope discrimination (δ^13^C), this ratio was quantified for each wine studied. Statistical analyses combining δ^13^C and sensory data highlighted that δ^13^C-values discriminated effectively between the most- and least-typical wines. In addition, Principal Component Analysis (PCA) revealed correlations between δ^13^C-values and truffle, undergrowth, and mint aromatic notes, three characteristics of the red Bordeaux wine aging bouquet. These correlations were confirmed to be significant using a Spearman statistical test. This study highlighted for the first time that vine water deficit positively relates to the perception of aging bouquet typicality, as well as the expression of its key aromatic nuances.

## Introduction

The balance between youthful fruitiness and tertiary aromas of fine aged wines within a complex, harmonious ensemble is particularly sought-after by wine professionals (Robinson and Harding, 2006; Jackson, 2009).

A significant mystery still surrounds the development of positive wine aging characteristics, usually called aging bouquet. Its chemistry and the factors responsible are not fully understood or well-characterized. Accordingly, deciphering the aging bouquet typicality of red wines remains one of the main current challenges in wine science. Recently, major advances have been made toward elucidating this complex olfactory concept, both from sensory and molecular perspectives. Specific aromatic properties (Picard et al., 2015a), as well as the contribution of several chemical markers (Picard et al., 2015b, 2016a), have been identified.

Moreover, it is well-established that the concepts of typicality and wine quality are closely linked with the notion of *terroir* (Jackson and Lombard, 1993; Sauvageot, 1994; Salette, 1997; Verdù-Jover et al., 2004; Casabianca et al., 2005; van Leeuwen and Seguin, 2006; Cadot et al., 2010, 2012). Nevertheless, even if the importance of *terroir* has been clearly identified in the conceptualization of the aging bouquet by wine professionals (Picard et al., 2015a), as well as in the perceived difference in mint aroma intensity (Picard et al., 2016b), no in-depth study had previously investigated how certain viticultural parameters conditioned the genesis of an aging bouquet after several years of bottle storage.

Among the various parameters of *terroir* that influence grape quality, the key role of vine water uptake conditions has repeatedly been emphasized (van Leeuwen and Seguin, 1994; van Leeuwen et al., 2004; Peyrot des Gachons et al., 2005; De Royer Duprè et al., 2014). More precisely, it has been shown in Bordeaux vineyards that a limitation in water supply (known as “water deficit”) is a key factor in explaining the vines' physiological and biochemical responses, leading to enhanced winemaking potential in the grapes (van Leeuwen et al., 2009). Water deficit occurs when vine transpiration and evaporation from the soil surface exceed incoming rainfall and irrigation (Lebon et al., 2003). In Bordeaux, all vineyards are dry farmed, so climatic factors are predominant in this study. In this process, soil water acts like a buffer: it compensates for climatic water deficits up to a certain threshold. When approximately 60% of the plant-available water reserves of the soil have been depleted, vines experience water deficit and trigger mechanisms, like stomatal closure, to save water (Pellegrino et al., 2006). The training system also influences the water balance by impacting light interception by the vines, which provides the energy for transpiration (van Leeuwen et al., 2010). Hence, vine water status is dependent on climate (variable from one vintage to another), soil (variable from one estate to another and even between plots on a given estate), and training system (variable from one estate to another; van Leeuwen et al., 2009). Training systems are rather homogeneous in high-quality Bordeaux wine producing vineyards (high-density, vertical-shoot positioned system) and are, therefore, unlikely to have a major influence on results in this study.

Due to the effect of carbon isotope discrimination during the photo-assimilation of CO_2_ (Farquhar et al., 1989; Gaudillère et al., 1999), the ^13^C/^12^C ratio measured in grape sugar at ripeness (usually expressed as δ^13^C) is considered an integrated physiological indicator for assessing the average vine water status during grape ripening (van Leeuwen et al., 2001; Gaudillère et al., 2002). Moreover, it has been shown that δ^13^C, measured in grape sugar at harvest, is well-correlated with the ethanol δ^13^C in wine produced from these grapes (Roßmann et al., 1996).

Recently, an easy-to-implement method based on isotope ratio measured by mass spectrometry coupled to an elemental analyzer (irm-EA/MS) was developed to evaluate wine carbon ^13^C isotope composition and validated using a set of wine samples produced from well-characterized vineyard blocks (Guyon et al., 2015). This analytical method highlighted a clear correlation between wine δ^13^C and the water status of the vines on which the grapes had grown. These results provided a relevant scientific basis for tracing the water status history of the vines from which aged red wines originated. Consequently, this strategy offered an appropriate tool for evaluating the impact of water vine uptake conditions on aging bouquet expression.

In this study, our aim was to investigate the link between vine water status and the sensory expression of aging bouquet typicality in mature red Bordeaux wines. It focused particularly on undergrowth, truffle, and mint notes, since their chemical composition had already been partly deciphered. Indeed, the direct contribution of dimethyl sulfide (DMS) to truffle and undergrowth attributes, as well as the overall complexity and balance of wine has been widely reported (Du Plessis and Loubser, 1974; Spedding and Raut, 1982; Segurel et al., 2004; Picard et al., 2015b). Moreover, it has already been shown that the potential of DMS (PDMS), susceptible to be chemically released by *S*-methyl methionine degradation (Segurel et al., 2004; Loscos et al., 2008) is dependent upon numerous *terroir* factors, including vine water status, grape maturity at harvest, and the yeast assimilable nitrogen content of must (Bell and Henschke, 2005; Dagan, 2006; Dagan and Schneider, 2012; De Royer Duprè et al., 2014). Concerning the mint aroma, a varietal effect on a wine's piperitone profile has recently been reported (Picard et al., 2016b) and the presence of *p*-menthane lactones, resulting from the same monoterpene limonene secondary biotransformation pathway as piperitone, was recently reported in wine (Picard et al., 2017), suggesting a common varietal origin for all these minty-terpenoid aromatic compounds.

After a sensory characterization of the aging bouquet typicality of some mature red wines, the bulk wine δ^13^C ratio was measured to estimate the vine water status during grape ripening. This parameter was then used to evaluate whether the sensory perception of aging bouquet typicality was related to the water status of the vines from which the wines originated. Finally, the impact of vine water status on the expression of “mint,” “undergrowth,” and “truffle” descriptors was also investigated.

## Materials and methods

### Chemicals

Carrier (Helium, 5.6 grade) and reference (carbon dioxide, 4.5 grade) gases were provided by Linde (Bassens, Bordeaux, France). Ethanol BCR 660 was obtained from the IRMM (Institute for Reference Materials and Measurements, Geel, Belgium) and tin cups for liquids (2.9 × 6 mm) from Elementar (France).

### Wines

#### First vertical tasting session

Twenty-one commercial red wines from three well-known Bordeaux appellations (Margaux, Pomerol, and Saint-Emilion), and three different estates (i.e. MX, P1, and SEM) were subjected to ortho- and retronasal sensory characterization. They were oak-barrel aged and produced between 1995 and 2013. All wines were tasted in 2015.

#### Second vertical tasting session

Twenty-one commercial red wines from four Bordeaux appellations (Margaux, Pomerol, Saint-Estèphe, and Saint-Emilion), and four different estates (i.e. MX, P1, P2, SE, and SEM) were subjected to ortho- and retronasal sensory characterization. They were oak-barrel aged and produced between 1995 and 2012. All wines were tasted in 2017.

Overall, the whole set of 42 wine samples was composed of five distinct series: Margaux (MX), Saint-Estèphe (SE), Saint-Emilion (SEM), and two different Pomerol (P1 and P2), each wine series originating from a single producer (Table 1).

**Table 1:** Characteristics of the 42 red Bordeaux wines tasted in 2015 and 2017 (appellation and vintage).

**Appellation**	**Vintage**	**Tasting year**
Margaux (MX)	1995/2000/2001/2008/2011/2013	2015
	2004/2005/2006/2009	2017
Pomerol (P)	P1 series: 1996/1998/2001/2004/2007/2010/2012	2015
	P2 series: 1995/1998/1999/2000/2001/2003/ 2005	2017
Saint-Emilion (SEM)	1995/1996/1997/2001/2003/2005/2008/2012	2015
	1998/1999/2000/2002/2004/2010	2017
Saint-Estèphe (SE)	1999/2000/2002/2012	2017

None of the wines included in the two tasting sessions presented premature oxidation character (i.e., prune or fig aromas) and they were all tasted within 30 min after the bottles were opened.

### Sensory analyses

#### Tasting conditions

Sensory analyses were performed as described by Martin and de Revel (2000). Samples containing about 20 mL wine were evaluated at 18°C in a dedicated room and in individual booths, using covered, black, ISO glasses (NF EN ISO 8589: 2007). The presentation order was randomized among the panelists, in a Latin Square arrangement. For all tasting sessions, wines were presented in a small range of vintages (i.e., within a 5-year period) to avoid any marked aging effect.

#### Wine professional panels

The first tasting session was conducted by a panel of 13 wine professionals (panel 1: 3 women and 10 men, age range: 31–75, mean age: 42 years). The panel for the second tasting only changed slightly (panel 2: 11 wine professionals, 3 women and 8 men, age range: 25–70, mean age: 40 years).

All the panelists consumed wine regularly, worked in the Bordeaux area, and had considerable experience in tasting Bordeaux wines. As both tasting sessions focused on their previous experience and their own mental representation of wine aging bouquet, the panelists did not undergo prior common training. They were informed about the context of the study but not the vintages, appellations, producers, or other wine characteristics.

The sensory performance of the two panels was assessed and confirmed by their ability to reproduce the same classification of two sets of five wines perceived as either good or poor examples of the wine aging bouquet typicality over a 10 months period (Picard et al., 2015a,b).

#### Experimental procedure

In both tasting sessions, the task of the professional panel consisted of a typicality assessment of the wine aging bouquet, based on a methodology developed during previous research about the Chardonnay wine concept (Ballester et al., 2005; Jaffré et al., 2011) and previously applied to the aging bouquet concept (Picard et al., 2015a). The wines were separated into three sets of seven wines (i.e., a same wine series encompassed only vintages belonging to a 5-year period). In each set, the order of presentation was randomized among the panelists. The instructions given were adapted from Ballester et al. (2005), as follows: “*Imagine that you have to explain the aging bouquet of red Bordeaux wines to someone. For each tasted wine, please answer the following question: do you think this wine is a good or a poor example for illustrating wine aging bouquet?”* Wine tasters answered on a 10 cm unstructured scale, ranging from “poor example” on the left to “good example” on the right (one scale per sample, one set of six wines per sheet). The score of each wine corresponded to “the typicality score” and was converted into a figure between 0 and 10.

Concerning sensory profiling, the intensity of the mint, truffle, and undergrowth aromatic descriptors were rated using a 10 cm unstructured scale, ranging from “odor not perceived” on the left to “very intense” on the right. The distance between the left end and the score-mark made by each panelist was measured and converted into a score between 0 and 10, indicating the sensory intensity of each aromatic attribute.

### Carbon isotope discrimination measured in wine samples

#### Instrumentation

δ^13^C was measured according to the methodology published by Guyon et al. (2015). Analyses were carried out using an elemental analyzer (EA, VarioMicroCube, Elementar, F-69623 Villeurbanne, France) coupled to a mass spectrometer for isotope ratio monitoring (irm-EA/MS, Isoprime/Elementar, F-69623 Villeurbanne, France). Ten μL wine samples were poured into tin capsules and injected into the oxidation tube (950°C) under helium (200 mL/min) and oxygen (30 mL/min) flux, at a reduction furnace temperature of 550°C. Combustion gases were dried and eluted on a specific column that physically retains the CO_2_ (60°C) and then releases it when the temperature is increased (210°C). An open split system was used to regulate gas withdrawal to the irm-MS and the current trap was set at 200 μA. The overall measurement duration was 600 s.

#### Isotope ratio computation

Measured masses m/z 44 and 45, corresponding to ^12^C^16^O_2_ and ^13^C^16^O_2_, respectively, were used to compute the^13^C/^12^C ratio, according to the formula: δ^13^C = ([(^13^C/^12^C)_sample_/(^13^C/^12^C)_standard_] − 1) × 1,000, expressed in % vs. PDB standard. Each analysis was duplicated and the results were an average of both measurements, with a variation coefficient below 0.3%.

### Statistical analysis

The typicality scores for the 42 red wines were statistically analyzed using the following procedure (Picard et al., 2015a). After validating the consensus among panelists by Principal Component Analysis (PCA) based on a wines × judges correlation matrix (data not shown), a two-way analysis of variance (ANOVA) was performed on each sensory data (i.e., typicality scores and descriptor intensity ratings) for assessing the discrimination ability of attributes. The judge factor was considered a random effect, while the wine factor was taken to be fixed. The *F*-ratio of the wine effect served as marker of the discrimination ability.

A Hierarchical Cluster Analysis (HCA), using the Ward method (dissimilarity criteria), was then applied to the wine typicality score. This methodology was used to assess the degree of similarity among wine samples and sort them into clusters, according to their aging bouquet typicality score. Wilcoxon non-parametric statistical tests (paired wine sample comparisons) were applied to both the typicality scores and δ^13^C measurements of the least and most typical wines.

All quantitative data obtained on the set of red Bordeaux wines were analyzed using PCA. For each wine, the correlation matrix included mean intensity values for each aromatic descriptor, typicality, and δ^13^C measurements.

Spearman product-moment correlations were also computed between δ^13^C-values, aging bouquet typicality score, and aromatic intensity ratings, at a significance level of α = 0.05.

All statistical analyses were conducted using XLSTAT software (Addinsoft, Paris, France, 2017).

## Results and discussion

Previous published data had not detected strict correlations between aging and bouquet typicality scores and wine age, although a vintage effect was highlighted (Picard et al., 2015b). As vintage quality is closely determined by the climatic conditions during vine development and grape ripening, particularly vine water status (van Leeuwen et al., 2009; van Leeuwen and Darriet, 2016), we hypothesized a potential impact of vine water deficit on aging bouquet development during bottle storage.

Principal Component Analysis (data not shown) was firstly used to validate the consensus among wine professionals on aging bouquet typicality. According to ANOVA, the effect of wine was significant for the typicality score (*F* = 6.426; *p* < 0.001), mint (*F* = 8.099; *p* < 0.005), truffle (*F* = 10.913; *p* < 0.001), and undergrowth (*F* = 5.861; *p* < 0.005) notes, indicating that these attributes were useful in characterizing aromatic differences among the wines.

The 42 wines studied during the two tasting sessions, in 2015 and 2017, were then clustered into three main groups according to the data obtained from the typicality task. An aging bouquet typicality gradient emerged, ranging from wines with the lowest (19 wines; typicality scores from 1.5 to 4.3) to wines with the highest average typicality scores (13 wines; typicality scores from 4.6 to 6.7). Between these two clusters, a group consisting of 10 wines was identified as “intermediates,” as their typicality scores varied drastically among wine professionals.

The next step was to investigate the existence of a correlation between aging bouquet expression in the wines studied and vine water status.

**Figure 1:**
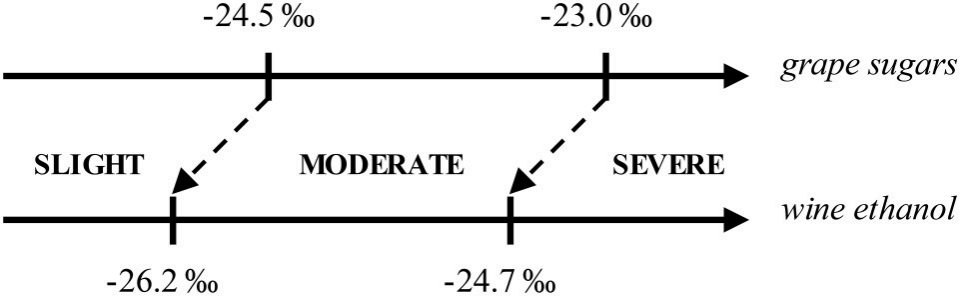
Relationship between δ^13^C-values and vine water deficit thresholds in grapes and wine, respectively. Three levels of vine water deficit are defined: slight water deficit (δ^13^C-values lower than −24.5% in grapes and −26.2% in wine); moderate water deficit (δ^13^C-values between −24.5 and −23.0% in grapes and −26.2 and −24.7% in wine); severe water deficit (δ^13^C-values higher than −23.0% in grapes and −24.7% in wine).

**Figure 2:**
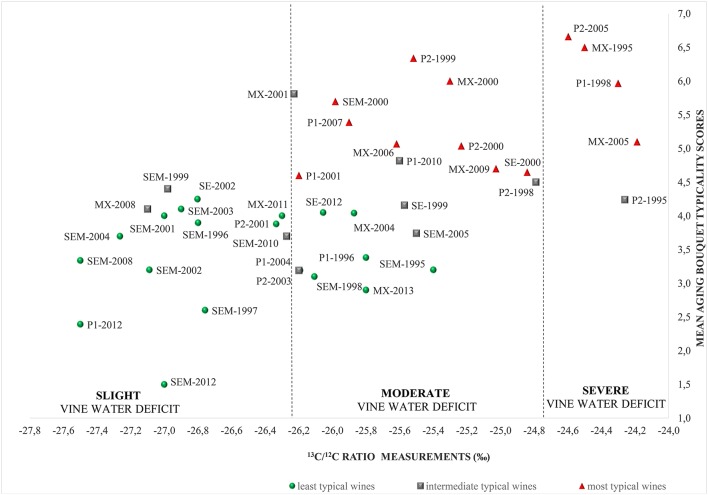
Relationship between aging bouquet typicality scores and δ^13^C representing the level of vine water deficit for the set of red Bordeaux wines considered (least, intermediate, and most typical wines represented by circles, squares, and triangles, respectively). Letters indicate the appellations (P, Pomerol; MX, Margaux; SE, Saint-Estèphe; SEM, Saint-Emilion). The following numbers indicate the vintage of each wine. The two Pomerol series are distinguished by numbers 1 and 2 (i.e., P1 and P2).

The bulk wine ^13^C/^12^C ratio was measured in all wines and the corresponding δ^13^C-values computed. Results ranged from −27.5 to −24.2%. Previous experiments conducted on grape must had identified a significant correlation between δ^13^C measured on grape sugar and water deficit (Gaudillère et al., 2002; van Leeuwen et al., 2009). Moreover, a study of the bioconversion of sugar to ethanol during the fermentation process revealed that δ^13^C is 1.7% lower in wine ethanol than grape sugar (Roßmann et al., 1996). Consequently, δ^13^C measured in bulk wine was a good indicator for evaluating vine water status during grape ripening. Accordingly, and relying on previously published data, three levels of vine water deficit were defined: slight water deficit when the δ^13^C-values were lower than −26.2%, moderate water deficit when δ^13^C was between −26.2 and −24.7%, and severe water deficit when δ^13^C exceeded 24.7% (Figure 1). All wines tasted were classified according to their δ^13^C ratio and typicality score (Figure 2). Overall, the results showed a link between aging bouquet expression and the corresponding vine water status. Most of the wines with a marked aging bouquet were produced in vintages where the vines were subjected to a moderate to severe water deficit (i.e., 1995, 1998, 2000, 2005, 2006, and 2009; δ^13^C > −25.6%; Figure 2). On the contrary, most of the wines in the group with the lowest typicality scores were produced in “wet” vintages with a weak to moderate water deficit (i.e., 1996, 1997, 1999, 2002, and 2008).

The statistical Spearman test showed that δ^13^C differentiated significantly between the most- and least-typical wines (*p* < 0.001, Table 2). Moreover, a significant positive correlation was observed between aging bouquet typicality scores and vine water deficit status, with significantly higher δ^13^C-values measured in wines with a high typicality score (Table 3). However, for some vintages, a wide dispersion was observed concerning the water status indicator. Even in the same vintage, different vine water regimes may exist in the four appellations studied, due to spatial variations in rainfall and/or soil water-retention capacity. This was the case, for example, in 1998: wines in the Pomerol series (P1 and P2) were associated with a severe water deficit (δ^13^C = −24.3 and −24.8%, respectively) whereas the Saint-Emilion wine was produced from vines facing a slight water deficit (δ^13^C = −26.11%). The same tendency was observed in 1999, when wines from Saint-Emilion (δ^13^C = −27%) were produced under slight water deficit conditions, while those from Saint-Estèphe and Pomerol had faced moderate water deficits (δ^13^C = −25.5 and −25.6%, respectively). The 2003 vintage was considered atypical, as high temperatures were associated with regular rainfall. Under these conditions, the vines were subjected to only moderate water deficit, as observed in the P2 Pomerol series (δ^13^C = −26.2%), thus confirming the results of a previous study (van Leeuwen et al., 2009).

**Table 2:** Range of aging bouquet typicality score and δ^13^C values obtained for the red Bordeaux wines studied, according to their correlation with aging bouquet typicality (least vs. most typical wines).

		**Most typical wines (*n* = 13)**	**Least typical wines (*n* = 19)**	*p*-values^*^
Typicality scores	Mean (±*SD*)	5.5 (±0.3)	3.4 (±0.4)	<0.0001
	Min	4.6	1.5	
	Max	6.7	4.3	
δ^13^C (%)	Mean (±*SD*)	−25.2 (±0.3)	−26.6 (±0.3)	<0.0001
	Min	−25.4	−27.5	
	Max	−24.2	−26.2	

* *P-Values indicate significant differences at p < 0.05 using a Wilcoxon non-parametric statistical test at a significance level of 95% for the aging bouquet typicality score and the δ^13^C-values between most- and least-typical wines*.

**Table 3:** Spearman product correlation parameters (correlation coefficient and *p*-value) obtained for the correlation between δ^13^C values and sensory intensity ratings (aging bouquet typicality score; truffle, mint, and undergrowth aromatic descriptors).

**Sensory descriptor**	***r***	***p*-values**	**Correlation**
δ^13^**C**			
Aging bouquet typicality	0.562	<0.001	Positive
Truffle	0.533	<0.001	Positive
Mint	0.413	0.005	Positive
Undergrowth	0.277	0.05	Slightly positive

However, within the Saint-Emilion series, the wine produced in 2003 had a low ^12^C/^13^C ratio (δ^13^C = −26.9%), corresponding to a slight vine water deficit, instead of the expected moderate value. Differences in δ^13^C within the same vintage are, at least partly, explained by differences in Soil Water Holding Capacity (SWHC). Average SWHC was higher in the Saint-Emilion vineyard (SEM) than the Pomerol, Margaux, or Saint-Estèphe vineyards, accounting for the less severe water deficits in most vintages in SEM. SWHC also varies from one plot to another. Hence, δ^13^C-values reported for the wines in this study reflected the average vine water deficit of the plots where the grapes in the final blend of the estate wine were harvested. This is one of the limitations of this study. Greater precision would have been possible if the wines had been produced from a single plot, which is rarely the case in the Bordeaux area.

**Figure 3:**
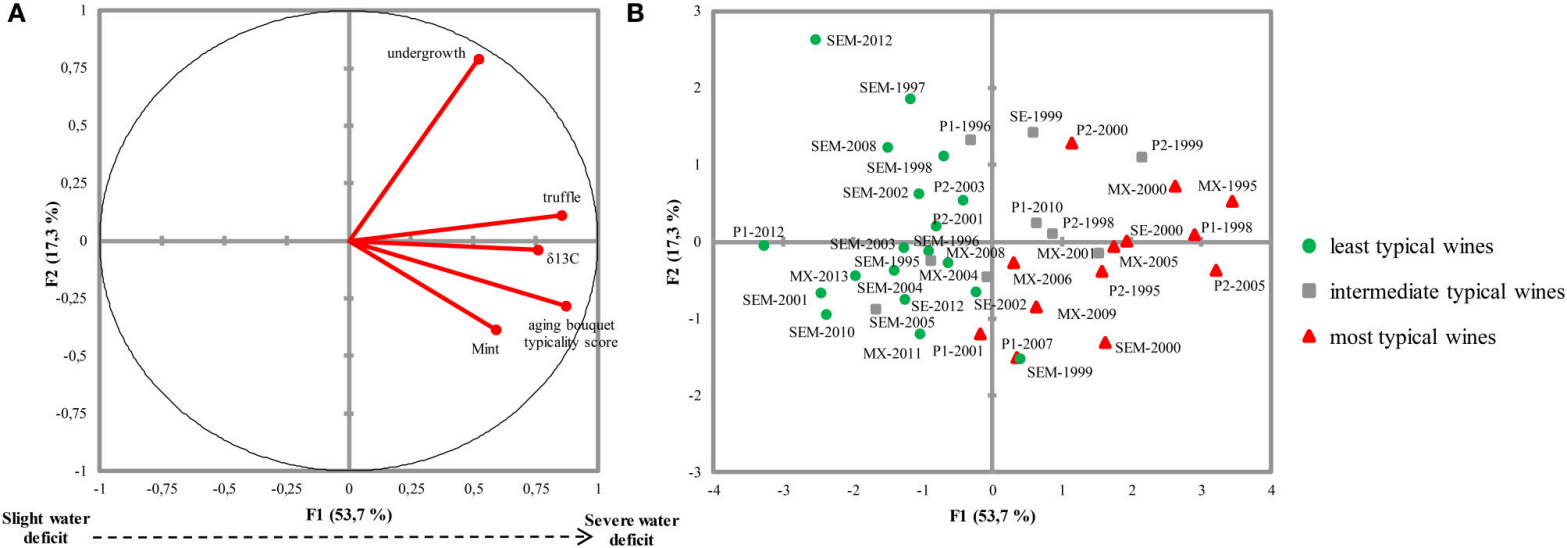
**(A)** PCA correlation circle of (percentage of total variability explained: 71%) presenting typicality scores, aromatic descriptors, and δ^13^C measurements and **(B)** the corresponding projection map of wines, classified according their typicality, obtained by HCA(least, intermediate, and most typical wines represented by circles, squares, and triangles, respectively). Concerning the wine codes of the map projection, letters indicate the appellations (P, Pomerol; MX, Margaux; SE, Saint-Estèphe; SEM, Saint-Emilion). The following numbers indicate the vintage of each wine. The two Pomerol series are distinguished by numbers 1 and 2 (i.e., P1 and P2).

Furthermore, the typicality scores and sensory intensities of mint, undergrowth, and truffle notes, as well as the ethanol ^13^C/^12^C ratio in the wines, were statistically processed using PCA, considering the first two components (Figure 3). The first principal component explained 53.7% of total variance and was positively correlated with all studied data. This result emphasized the good correlation between the typicality score and δ^13^C-values. Moreover, the loading plots for mint and truffle intensity ratings were projected on the same positive side of the first axis and were both close to the typicality score and δ^13^C loading plots. These two aromatic notes were thus well-correlated with the typicality of the wine aging bouquet and, more importantly, with the ^13^C isotope distribution in the wines. Although the undergrowth note was also projected on the positive side of the first axis, its position was close to the correlation circle, indicating that the impact of δ^13^C on its expression was less clear.

These results were confirmed using the Spearman correlation test: δ^13^C correlated significantly and positively with truffle and mint notes, while it only marginally correlated with the undergrowth note (Table 3).

Vintage quality in Bordeaux is known to be related to vine Water Deficit Status Index (WDSI), calculated between veraison and ripeness. The winemaking potential of the grape will suffer both in the case of no water deficit as in the situation of excessive water stress (van Leeuwen et al., 2009). The ideal water uptake conditions for producing high quality grapes for the production of red wine corresponds to a moderate water deficit, preferably starting early in the season (i.e., before veraison). Such an early water deficit has a greater effect on the reduction of berry size than late water deficit (Ojeda et al., 2002). However, the impact of vine water status on the development of the aging bouquet in red Bordeaux wines, as well as on several of its key aromatic notes, had not previously been described. Interestingly, our results revealed that the vine water deficit level significantly impacted not only overall aging bouquet typicality, but also mint, truffle, and, to a lesser extent, undergrowth aroma perception. These observations confirm several previous studies, which, although not dealing with aroma expression in wines, also showed a positive impact of a moderate water deficit on grapes quality potential, through an increase in phenolic compounds (in particular anthocyanins) and grape sugar concentrations (Duteau et al., 1981; Matthews and Anderson, 1988; van Leeuwen and Seguin, 1994; Ojeda et al., 2002). Peyrot des Gachons et al. (2005) reported that mild water deficit increases the concentration of volatile thiol precursors in Sauvignon blanc, while severe water deficit has a negative impact. Moreover, in a study comparing soil and climate effect on grape ripening, it was shown that grape quality potential was highest on clayey soils, because these induced early but moderate water deficits in most vintages (van Leeuwen et al., 2004).

Overall, these new advances support the idea that the *terroir*—and particularly vine water status—plays a key role in the corresponding molecular marker levels in wine and thus, influences the subsequent development of the related aromatic notes during positive wine aging.

## Conclusion

This study provides new insights into the influence of vine water status on aging bouquet expression in fine red Bordeaux wines. A recently-developed method, based on carbon-isotope discrimination measured in wine ethanol, applied to a large set of aged red wines from five different Bordeaux vineyards, confirmed the impact of vine water deficit on the development of the aging bouquet and, more precisely, the relevant impact of a moderate-to-severe water deficit on mint and truffle aroma expression.

Further work is still required to obtain a more comprehensive understanding of the links between water deficit and the aging bouquet on a molecular level. Following the recent identification of several molecular markers responsible for truffle and mint notes in wines with an aging bouquet, it would be relevant to perform a quantitative analysis of these specific aroma compounds, as well as their precursors, in monovarietal wines from various vintages and different soils, in order to investigate the relationship with vine water status. This next step is necessary to unravel the mechanisms that explain the biochemical pathways involved in this relation.

## Ethics statement

Our current work is only based on physicochemical analyses of commercial wines samples. It does not concern any sample issued from animal or human biological sources and, accordingly, does not require any ethics review process or written informed consents. Concerning the wine tastings, all wine professionals were consented and informed about the context of the study.

## Author contributions

All authors listed have made a substantial, direct and intellectual contribution to the work, and approved it for publication.

### Conflict of interest statement

The authors declare that the research was conducted in the absence of any commercial or financial relationships that could be construed as a potential conflict of interest.
